# A novel Cuproptosis-related LncRNA signature to predict prognosis in hepatocellular carcinoma

**DOI:** 10.1038/s41598-022-15251-1

**Published:** 2022-07-05

**Authors:** Genhao Zhang, Jianping Sun, Xianwei Zhang

**Affiliations:** 1 Department of Blood Transfusion, First Affiliated Hospital of Zhengzhou University, Zhengzhou, China; 2 Department of Pathology, Zhengzhou YIHE Hospital, Zhengzhou, China; 3 Medical School, Huanghe Science and Technology University, Zhengzhou, China

**Keywords:** Cancer microenvironment, Cancer therapy, Tumour biomarkers

## Abstract

Increased intracellular toxicity due to an imbalance in copper homeostasis caused by copper ion accumulation could regulate the rate of cancer cell growth and proliferation. The goal of this study was to create a novel Cuproptosis-related lncRNA signature that may be utilized to predict survival and immunotherapy in HCC patients. Cuproptosis-associated lncRNAs and differentially expressed lncRNAs between HCC tumor tissue and normal tissue were discovered first. By LASSO-Cox analysis, the overlapping lncRNAs were then utilized to build a Cuproptosis-associated lncRNA signature, which might be used to predict patient prognosis and responsiveness to immune checkpoint blockade (ICB) therapy. Differences in the infiltration of immune cell subpopulations between high and low-risk score subgroups were also analyzed. Moreover, a nomogram based on the Cuproptosis-associated lncRNA signature and clinical features was developed and demonstrated to have good predictive potential. Finally, qRT-PCR was performed in HerpG2 and MHCC-97H cell lines to explore whether these lncRNAs were indeed involved in the process of Cuproptosis. In summary, we created a prognostic lncRNA profile linked to Cuproptosis to forecast response to immunotherapy, which may provide a new potential non-apoptotic therapeutic perspective for HCC patients.

## Introduction

As a catalytic cofactor for essential enzymes involved in the regulation of energy conversion, iron collection, oxygen transport, and intracellular oxidative metabolism, the presence of appropriate amounts of copper in cells is irreplaceable for life^1^. The level of copper concentration in cells is regulated by metabolic requirements and variations in the cellular environment. Too little or too much copper can cause significant damage to cellular physiology, both in bacteria and in human cells. Imbalances in copper metabolism can seriously affect the development of the heart and central nervous system, and have a definite impact on the normal metabolism of the liver^2^. Imbalances in copper homeostasis caused by genetic variants can even lead to life-threatening diseases, such as Wilson's^3^ and Parkinson's diseases^4^. Moreover, decreased serum copper concentration has been reported to be associated with the development of endometrial cancer^5^, and an imbalance in copper homeostasis has also been observed in the progression of head and neck cancers^6^. Considering the double-edged function of copper, which is an essential enzyme cofactor but also produces toxicity that causes cell death, copper is expected to be a new therapeutic target used to specifically kill cancer cells by increasing intracellular copper accumulation^7^. Copper has been reported to be associated with resistance to platinum-based antitumor compounds and can be used as a radiotherapeutic agent in the combination treatment of cancer^8^. Copper can also strengthen the anti-tumor effect in patients by binding to Disulfiram (DSF)^9^. Excitingly, copper has been reported to be involved in a new mode of cell death, which was named "Cuproptosis"^10^. Copper can directly bind to lipid acylated elements of the tricarboxylic acid (TCA) cycle, inducing the onset of Cuproptosis, which leads to proteotoxic stress and finally to cell death in a manner that is not dependent on the apoptotic pathway. These all indicate the great potential of copper in antitumor therapy for cancers that are naturally resistant to apoptosis.

Long non-coding RNAs (lncRNAs) of only 200 nucleotides in length have been reported to be involved in a variety of tumorigenesis and progression by regulating the biological behavior of cancer cells, especially in hepatocellular carcinoma (HCC). A large amount of evidence suggests that lncRNAs can be involved in HCC progression, recurrence, and immunotherapeutic response by regulating multiple epigenetic pathways such as hypoxia^11^, m6A methylation^12^, ferroptosis^13^, autophagy^14^ and energy metabolism^15^. Few studies have reported the functions and roles of lncRNAs in the process of Cuproptosis, and it is important to gain insight into their role in the prognostic prediction of HCC patients. Here, we created and validated a potential profile based on Cuproptosis-associated differentially expressed lncRNAs (DE-lncRNAs) for prognostic prediction in HCC patients.

## Materials and methods

### Public data collection and identification of Cuproptosis-associated DE-lncRNAs

Transcriptome expression data of HCC patients were obtained from the TCGA-LIHC (https://portal.gdc.cancer.gov/) databases. Six patients without complete survival data were excluded. To compensate for the lack of TCGA normal samples, 110 normal liver tissue samples from the GTEx database (https://xenabrowser.net/datapages/) were used. The log2 (FPKM + 1) transformation was used to normalize the transcriptome data. The batch effects between TCGA and GTEx normalized data were corrected by ComBat of the R package ‘’SVA’’. DE-lncRNAs between normal and HCC tissues were screened out with cut-off criteria of P-value of < 0.05 and |log_2_FC| ≥ 1. Then, a total of 365 HCC patients with complete survival data were included in the follow-up analysis. Patients were randomly divided into training and testing groups according to the ratio of 3:2, and their basic information was shown in Table 1. Ten Cuproptosis-related genes (including FDX1, LIPT1, DLD, LIAS, DLAT, PDHA1, PDHB, MTF1, GLS, and CDKN2A) were obtained from a previous study^10^ and subsequently explored for their expression and prognostic value in HCC. To discover the Cuproptosis-related lncRNAs, Pearson correlation analysis was used to evaluate the correlations between the expression levels of the ten genes and those of lncRNAs. |Pearson correlation coefficient| > 0.4 and P-value of < 0.001 were the requirements. Overlapping lncRNAs were considered Cuproptosis-related DE-lncRNAs.

**Table 1:** Clinical characteristics of HCC patients involved in the study.

	Training cohort (N = 218)	Testing cohort (N = 147)	TCGA cohort (N = 365)
**Gender**			
Male	175	102	119
Female	73	45	246
**Age**			
≤ 60 years	111	63	173
> 60 years	107	84	192
**Grade**			
G1/2	136	76	230
G3/4	79	49	130
Unknown	3	2	5
**TNM stage**			
I/II	155	99	254
III/IV	52	35	87
Unknown	11	13	24
**Vascular invasion**			
Yes	66	40	106
No	125	80	205
Unknown	27	27	5
**Recurrence with tumor**	69	53	122
Tumor free	102	60	161
Unknown	47	34	82
**Cirrhosis**			
With	40	27	68
Without	89	53	141
Unknown	89	67	156

### Creation of Cuproptosis-associated lncRNA signatures

Univariate Cox regression with a P-value of < 0.05 was performed to screen lncRNAs related to patients’ prognosis among the Cuproptosis-related DE-lncRNAs. Then a Cuproptosis-related lncRNA signature was created by performing the least absolute shrinkage and selection operator (LASSO) Cox regression and multivariate Cox regression model in the training cohort. The multivariate Cox relapse coefficient (β) was used to generate a risk score founded on the notion of directly mixing the equation below with lncRNA expression levels. Risk score = ∑iCoefficient (lncRNAi)*Expression (lncRNAi). Time-dependent receiver operating characteristic (time-ROC) curves analysis, Kaplan–Meier survival analysis, and a cox relative risks relapse research were used to analyze the prognostic signature's predictive control and autonomy. In addition, to separate the altogether cautious GO and KEGG items with FDR < 0.05, gene set enrichment analysis (GSEA) was done between the high and low-risk scores groupings in the Metascape database^16^. Finally, a nomogram model was built to investigate the coefficient prediction efficacy of this Cuproptosis-related lncRNA in the TCGA dataset.

### Tumor-infiltrating immune cells and Genetic alterations analysis

The TIMER^17^ and xCELL^18^ databases were used to determine the abundance ratios of tumor-infiltrating immune cells in HCC immune microenvironment. HCC patients' mutation data were obtained from TCGA, and changes in genetic variations across distinct subgroups were evaluated using the R package "maftools."

### Cuproptosis cell model construction and qRT-PCR assay

HerpG2 and MHCC-97H cells were bought from the Shanghai Institute of Cells' Cell Bank (Shanghai, China) and grown in a prescribed DMEM medium (Sangon Biotech, China) with 10% fetal bovine serum (FBS, Sangon Biotech, China) at 37 °C with 5% CO^2^. According to the previous study^10^, a 2-h pulse treatment with 100 nM elesclomol (+ 1 µM CuCl_2_ in media) was performed to promote the occurrence of Cuproptosis in HerpG2 and MHCC-97H cells. After 24 h, cells were harvested and lysed. Total RNA was isolated using TRIzol reagent and mRNA was reverse transcribed using PrimeScript RT Master Mix Synthesis Kit (Sangon Biotech, China). Changes in the expression of these Cuproptosis-related lncRNAs before and after drug treatment were detected by qRT-PCR. Table 2 listed the primer sequences.

**Table 2:** The sequences of the qRT-PCR primers used in this study.

Gene	Forward primer	Reverse primer
AC138904.1	CTGCCAATTGCCAAAGGGTC	CCCCACACTTCCTGCGTAAT
AC099329.2	AAAACTCGAAACGTGTGCCG	GTGAATGGGGACTCACCCTG
DEPDC1-AS1	TCGCTCCTCATAGCGAGTCT	TGACTTCCTTATCCGCTCCC
GIHCG	CGGAGGCGATTGACCGTTAT	AGAGCCCTCACGACCCTTTA
AC145343.1	TTCTTGCCCGCCTGATGAAT	ACCGTAACACGCCACATCTT
DNMBP-AS1	TCGCGGACACATGAAGATGG	TGGGTGATGACTGAGGTCAC
GAPDH	GGAGCGAGATCCCTCCAAAAT	GGCTGTTGTCATACTTCTCATGG

### Statistical analysis

The independent-samples t-test was used to analyze the quantitative variables. ROC curve analysis and Kaplan–Meier survival analysis were used to analyze the efficacy of R software in predicting survival outcomes (version 4.0.3). The link between a prognostic classifier and survival outcomes, as well as other clinical parameters, was investigated using a Cox proportional model. When the P-value was less than 0.05, the results were considered statistically significant.

## Results

### Expression of Cuproptosis-associated Genes in HCC

**Figure 1:**
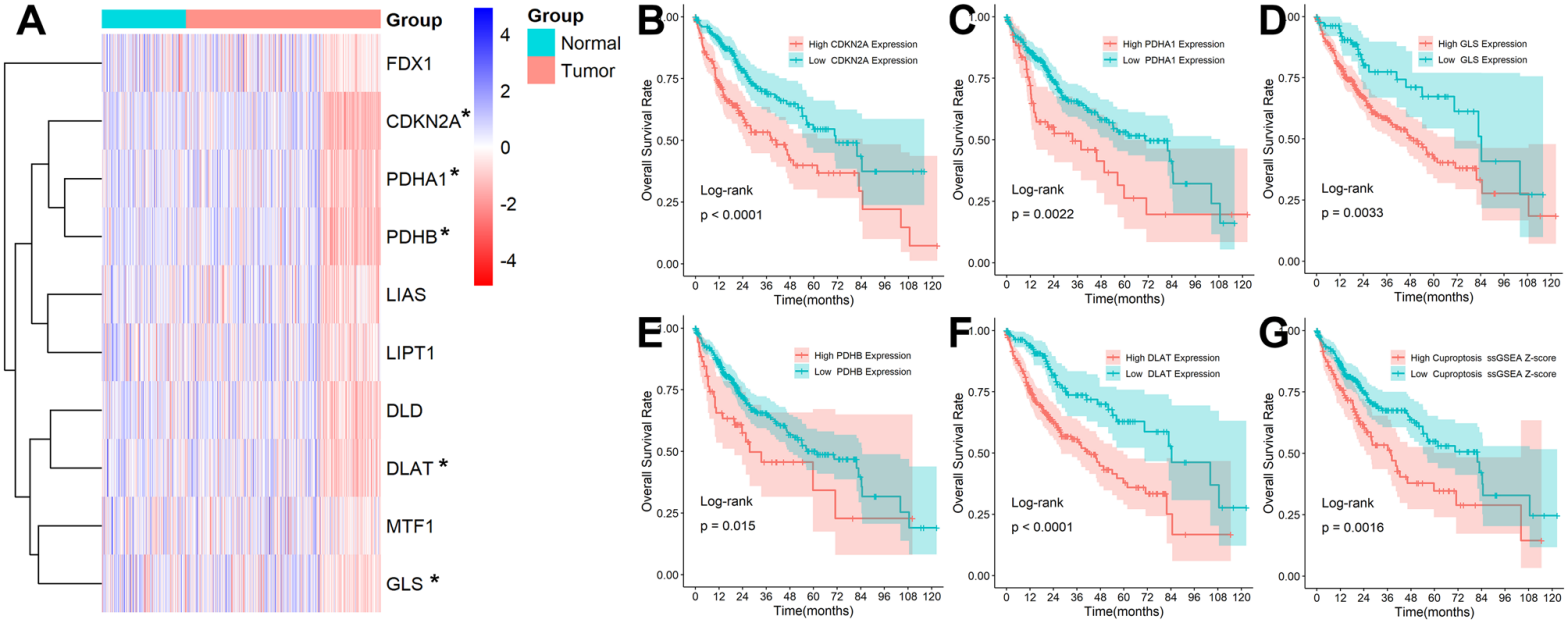
Expression of Cuproptosis-associated Genes in HCC. (**A**) Expression levels of ten genes in normal tissues and HCC. A prognostic value analysis of CDKN2A (**B**), PDHA1 (**C**), GLS (**D**), PDHB (**E**), DLAT (**F**), and Cuproptosis Z-scores (**G**).

As shown in Fig. 1A, half of the ten Cuproptosis-related genes, including DLAT, PDHA1, PDHB, GLS, and CDKN2A, differed significantly between normal and HCC tissues, and the high expression of these five genes was also closely associated with poor prognosis in HCC patients (Fig. 1B–F). Furthermore, the results of the ssGSEA analysis showed that patients with higher Cuproptosis Z-scores had shorter survival times (Fig. 1G). These all suggested the involvement of Cuproptosis in the prognostic development of HCC patients.

### Identification of Cuproptosis-associated DE-lncRNAs

**Figure 2:**
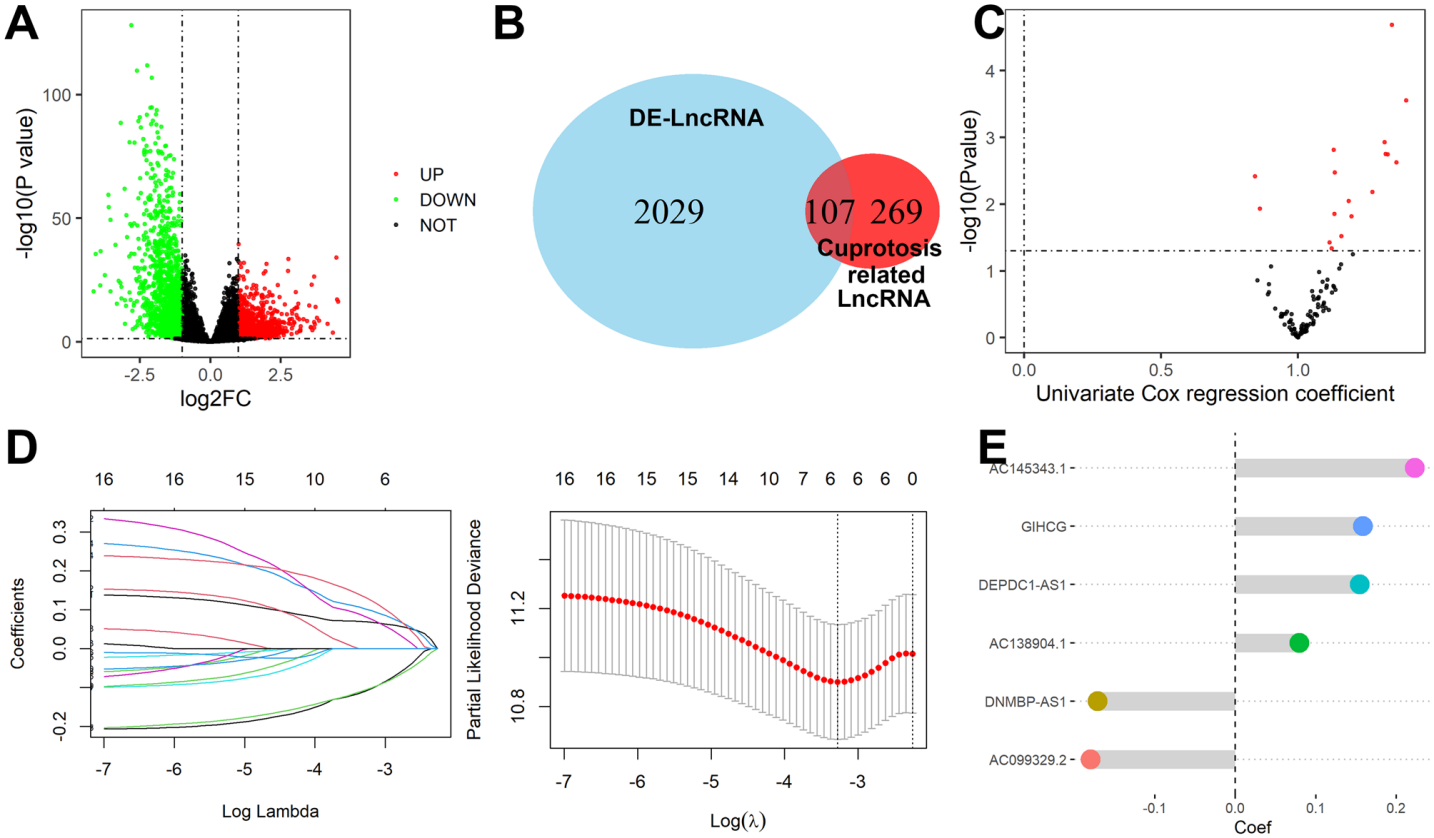
Identification of Cuproptosis-associated DE-lncRNAs. (**A**) DE-lncRNA in TCGA and GTEx database. (**B**) Cuproptosis-related lncRNA by Pearson correlation analysis. (**C**) 107 overlapping lncRNA were considered Cuproptosis-associated DE-lncRNAs. (**D**) lncRNAs screened by the LASSO-Cox regression model. (**E**) The multivariate Cox relapse coefficient.

A total of 763 up-regulated and 1373 down-regulated lncRNA were identified as DE-lncRNA in TCGA and GTEx database as shown in Fig. 2A, and 376 lncRNA were screened out as Cuproptosis-related lncRNA by Pearson correlation analysis. The 107 overlapping lncRNA were considered Cuproptosis-associated DE-lncRNAs (Fig. 2B).

### Creation and validation of Cuproptosis-associated lncRNA

**Figure 3:**
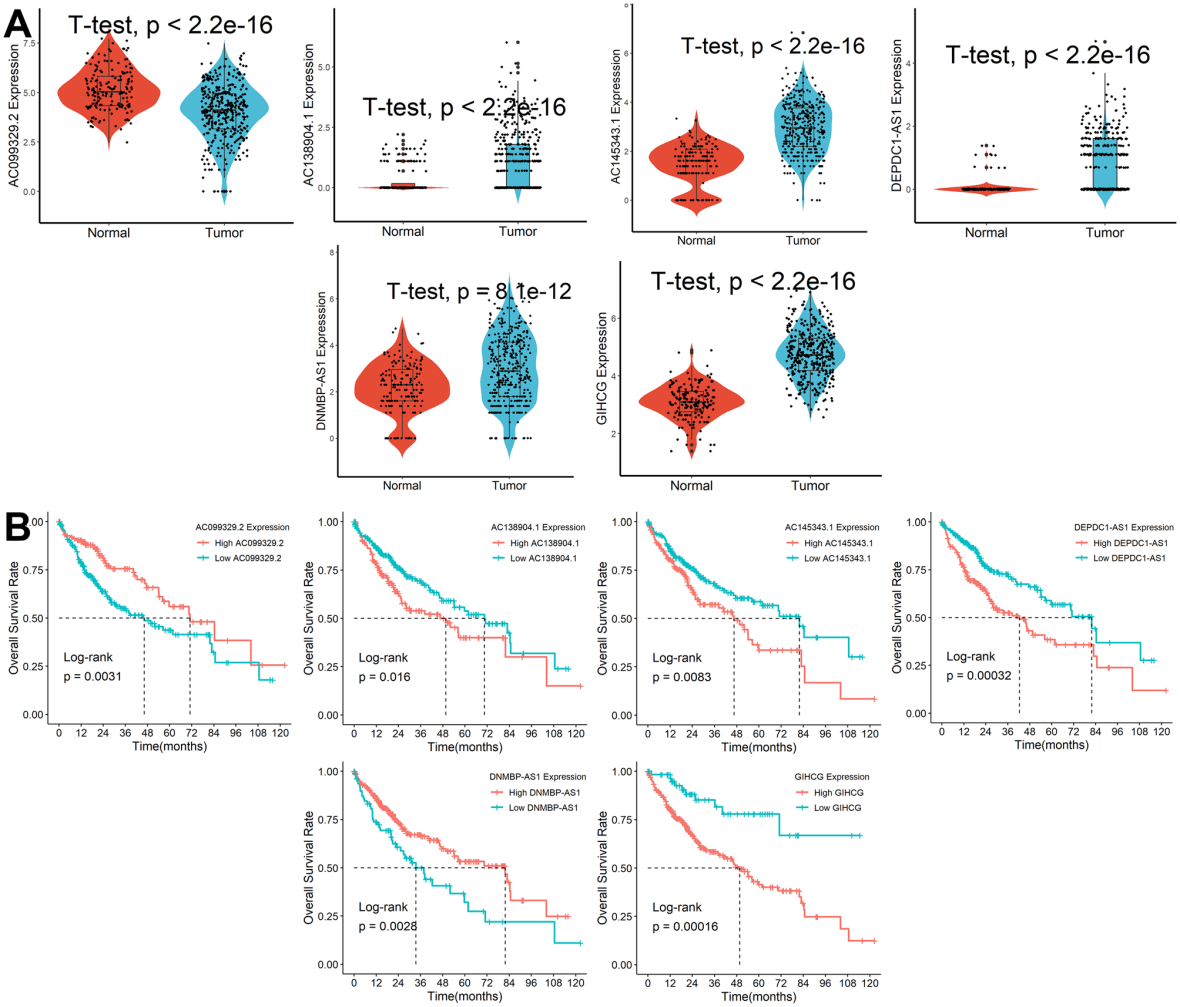
Expression and prognostic significance of the 6 lncRNAs. (**A**) Expression levels in tumor and normal tissues. (**B**) Prognostic value analysis.

**Figure 4:**
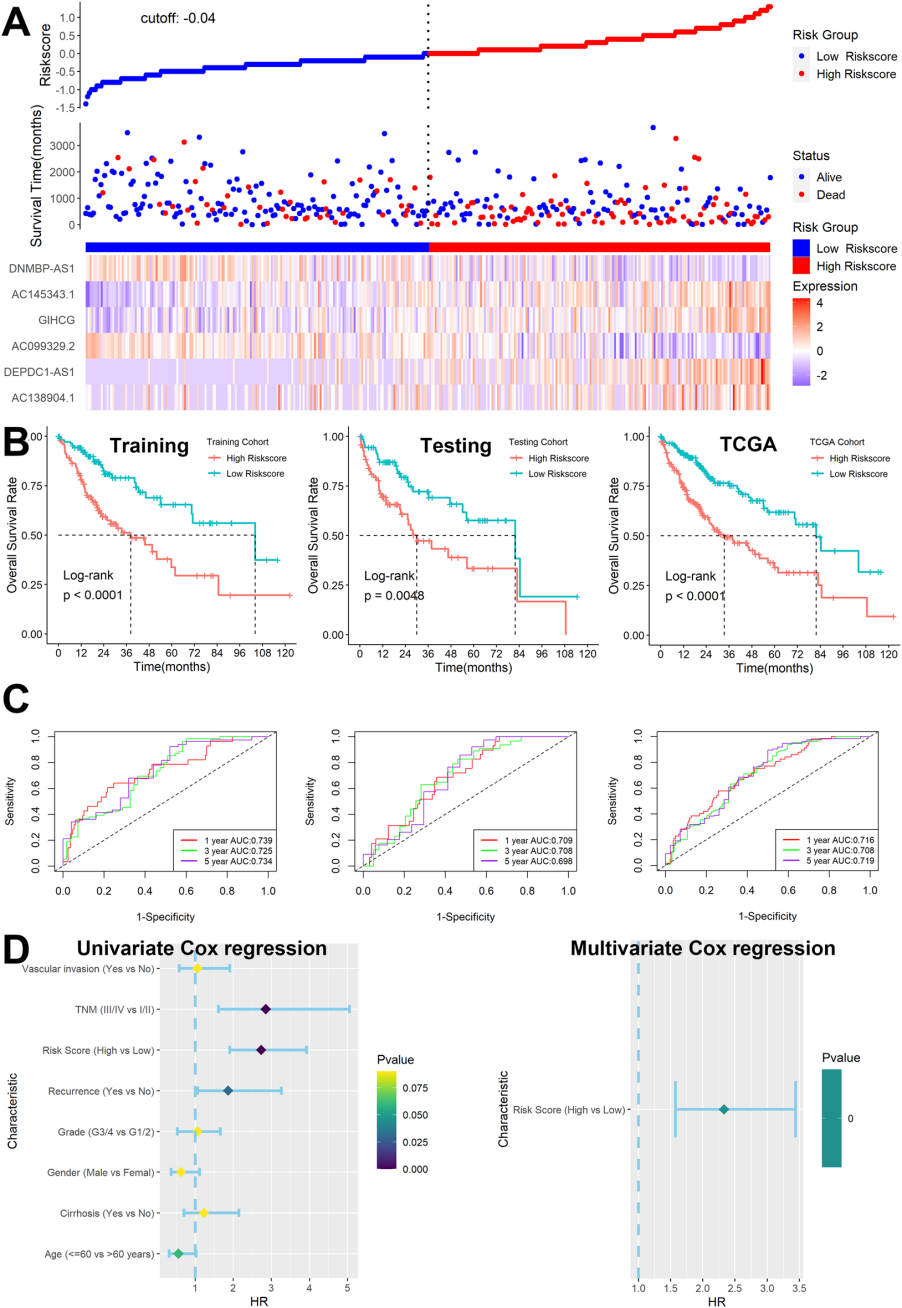
Construction of Cuproptosis-associated lncRNA signature in TCGA. (**A**) The distribution of risk scores, OS status, and expression profiles of the six lncRNAs. (**B**) Patients with higher risk scores had significantly decreased survival outcomes in training, testing, and TCGA cohorts. (**C**) ROC analysis in the three cohorts. (**D**) The Cuproptosis-associated lncRNA signature was shown to be an independent risk factor for patients' overall survival in TCGA.

A total of 16 candidate lncRNA with a P value less than 0.05 were identified to be associated with the patient's prognosis (Fig. 2C) and further screened by the LASSO-Cox regression model (Fig. 2D). A six-lncRNA signature was finally created by a multivariate Cox proportional model (Fig. 2E). Risk score = AC138904.1 * 0.07981 − AC099329.2 * 0.17935 + DEPDC1-AS1 * 0.15452 + GIHCG * 0.15857 + AC145343.1 * 0.22327 − DNMBP-AS1 * 0.17083. We found that all of the six lncRNAs were differentially expressed between tumor and normal tissues (Fig. 3A) and were closely associated with patients’ prognosis (Fig. 3B). After assessing the risk score for each HCC patient using the above formula (Fig. 4A), we found that patients with lower risk scores had a better survival outcome in both the training cohort, the testing cohort, and the TCGA cohort (Fig. 4B). According to time-ROC analysis, this signature has a great predictive ability, with AUCs of 0.739, 0.725, and 0.734 in the training cohort, 0.709, 0.708, and 0.698 in the testing cohort, and 0.716, 0.708, 0.719 in TCGA cohort at one, three, and five years, respectively (Fig. 4C). Finally, we found that this feature could be an independent prognostic factor for HCC patients after adjusting for other clinical characteristics (Fig. 4D).

### Cuproptosis-related lncRNA signature was related to the progression of HCC

**Figure 5:**
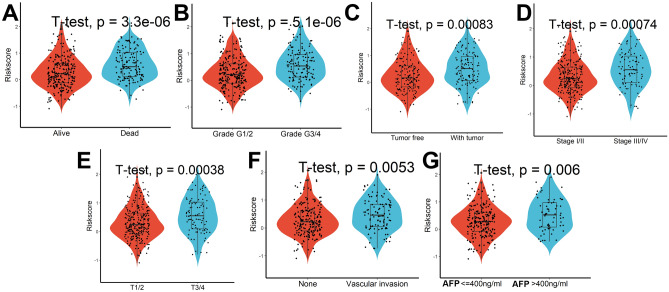
Relationship between Cuproptosis-related lncRNA signature and clinical characteristics. Analysis of differences in risk scores between patients with different survival status (**A**), grade (**B**), recurrence status (**C**), TNM stage (**D**), T stage (**E**), vascular invasion (**F**), and levels of AFP (**G**).

**Figure 6:**
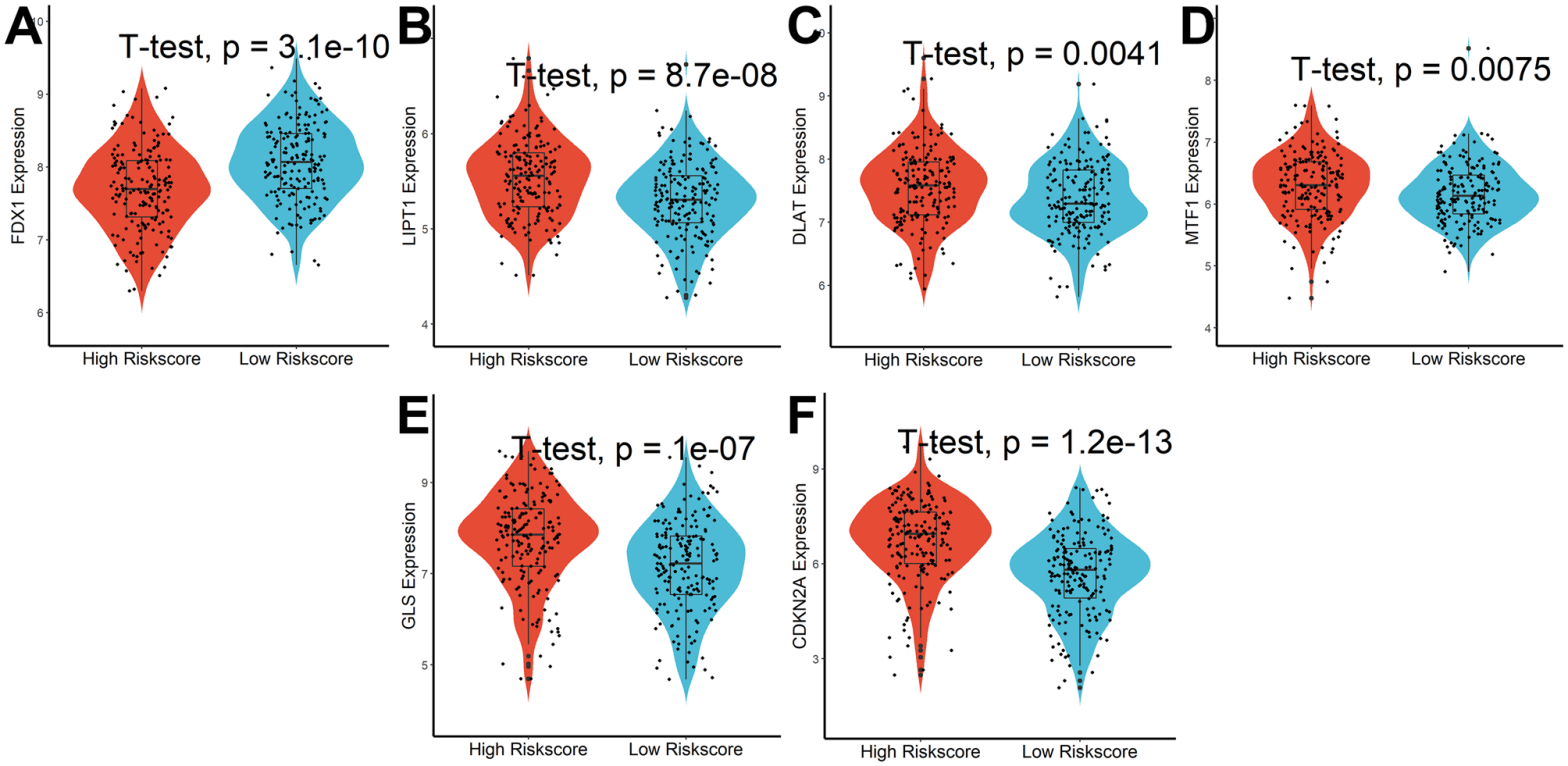
Analysis of differences in Cuproptosis-related genes between patients in high- and low-risk score groups. (**A**) FDX1, (**B**) LIPT1, (**C**) DLAT, (**D**) MTF1, (**E**) GLS, and (**F**) CDKN2A.

During follow-up, we found that patients who were dead had higher risk scores than those who were alive (Fig. 5A). Moreover, patients with more advanced clinical stages, such as later Grade (Fig. 5B), recurrence (Fig. 5C), later TNM stage (Fig. 5D), later T stage (Fig. 5E), vascular invasion (Fig. 5F), and high levels of Alpha-fetoprotein (AFP, Fig. 5G) had higher risk scores. All of these indicated that patients with lower risk scores had significantly improved survival results. Finally, we found that patients in the high-risk scores group had lower FDX1 levels (Fig. 6A) and higher LIPT1, DLAT, MTF1, GLS, and CDKN2A levels (Fig. 6B–F) when compared with patients in low-risk scores group.

### Functional enrichment analysis

**Figure 7:**
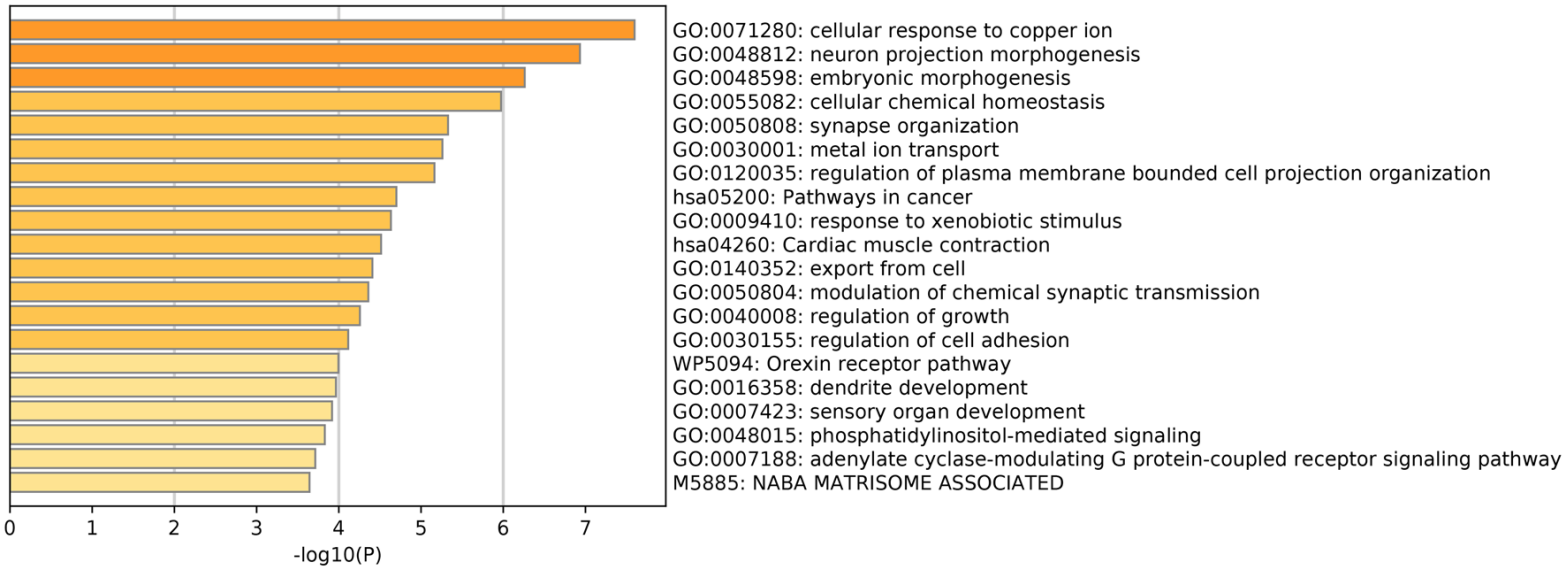
Enrichment analysis in the Cuproptosis-related lncRNA model.

Differentially expressed genes (DEGs) between high- and low-risk score subgroups were first explored with cut-off criteria of P-value of < 0.05 and |log_2_FC| ≥ 1 and then were used to identify the altogether cautious GO and KEGG items by GSEA in Metascape database. As shown in Fig. 7, these DEGs were mainly enriched in copper-related biological processes.

### Genetic alterations and immune infiltrate analysis

**Figure 8:**
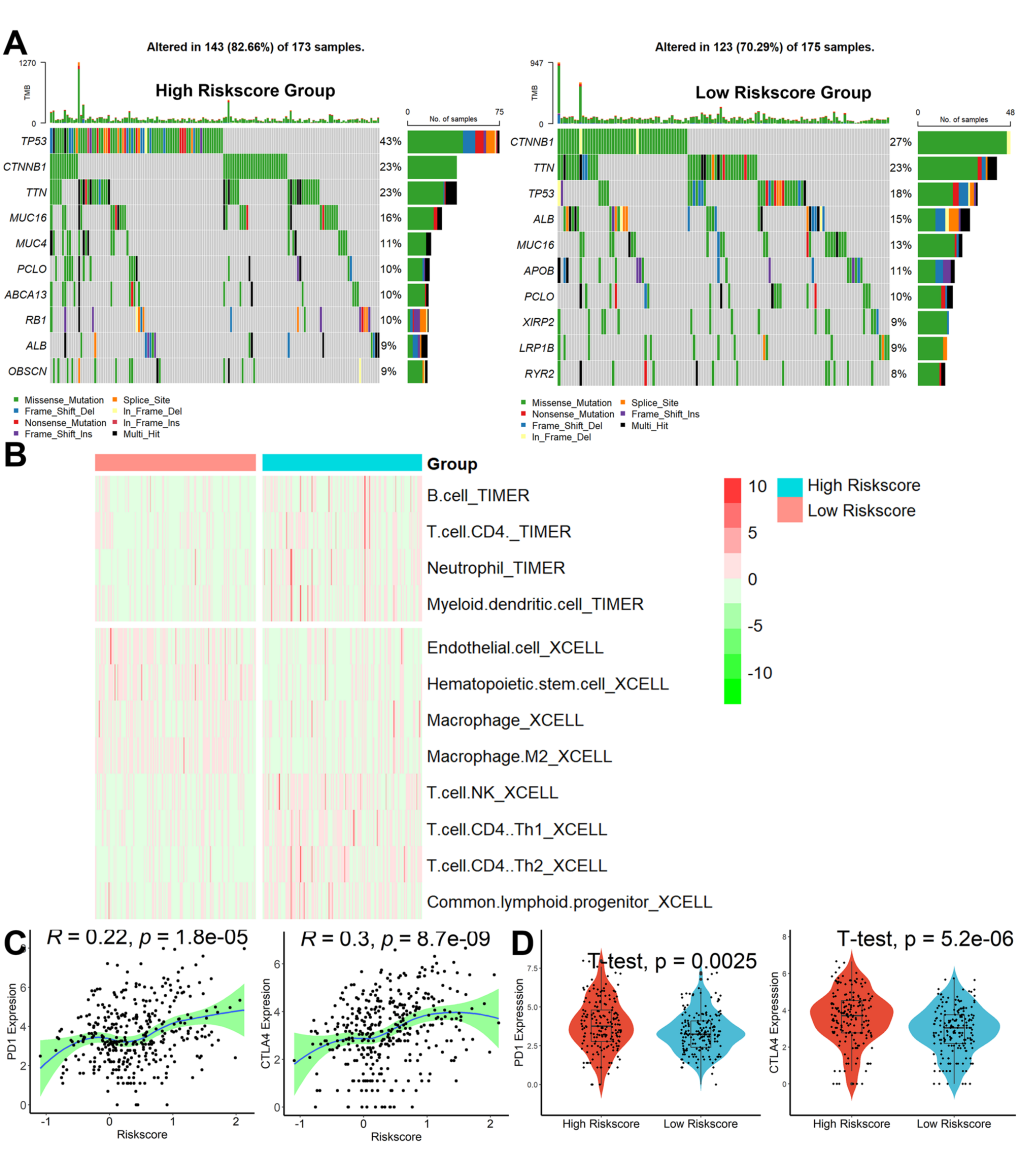
Genetic alterations and Immune Infiltrate Analysis. (**A**) Top 10 most substantially changed genes in the high- and low-risk score subgroups. (**B**) A heatmap of all substantially distinct immune cells between the high- and low-risk score subgroups. (**C**) Correlation analysis between risk scores and PD1, CATL4 expression. (**D**) Analysis of differences between risk scores and PD1, CATL4 expression.

According to the findings of genetic alteration analysis, the mutation rates of the top 10 most substantially changed genes were significantly different between the high- and low-risk score subgroups, as shown in Fig. 8A. In addition, as shown in Fig. 8B, we create a heatmap of all substantially distinct immune cells. The results of TIMER showed that infiltration levels of B cells, CD4+ T cells, neutrophil cells, and myeloid dendritic cells were remarkably higher in the high-risk score group. The results of XCELL showed that infiltration levels of NK cells, CD4+ Th1 cells, CD4+ Th2 cells, and common lymphoid progenitor cells were significantly increased while endothelial cells, hematopoietic stem cells, and macrophage cells were remarkably decreased in high-risk score group. Risk scores were significantly correlated with PD1 and CTLA4 expression (Fig. 8C), with patients possessing high-risk scores having higher levels of PD1 and CTLA4 expression (Fig. 8D), indicating that these patients may be more suitable for immunosuppressive therapy.

### Building a nomogram model

**Figure 9:**
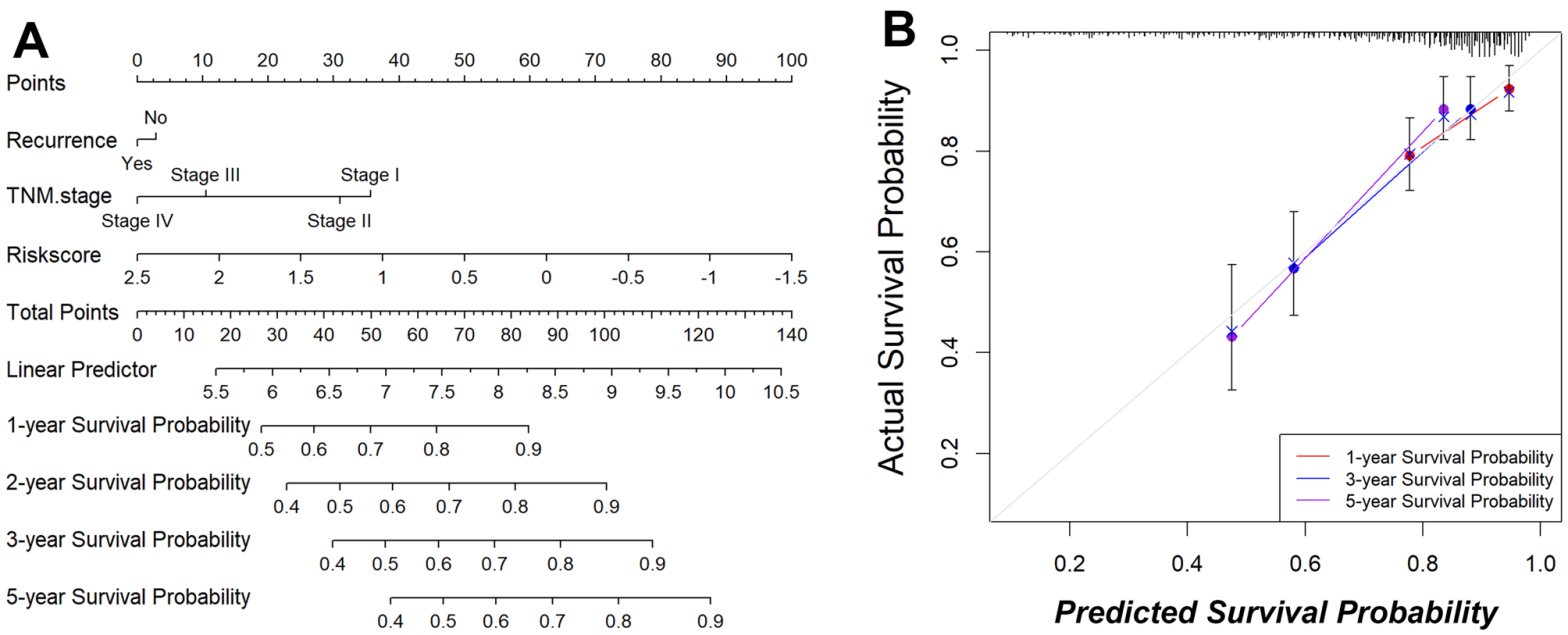
The predictive significance of the Cuproptosis-associated lncRNA signature was verified in the nomogram model. (**A**) Nomogram combining the six Cuproptosis-associated lncRNA signatures. (**B**) Calibration plots of 1-, 2-, and 3-year survival probabilities.

Using a nomogram model in the TCGA dataset, the coefficient prediction efficacy of this Cuproptosis-associated lncRNA signature was investigated, and the findings revealed that the nomogram could help us provide a quantitative approach for appropriately predicting the 1-, 3-, and 5-year survival rates (Fig. 9A). The calibration curves demonstrated that the predicted and actual 1-, 3-, and 5-year survival rates were all within a proper range (Fig. 9B).

### qRT-PCR assay in Cuproptosis cell model

**Figure 10:**
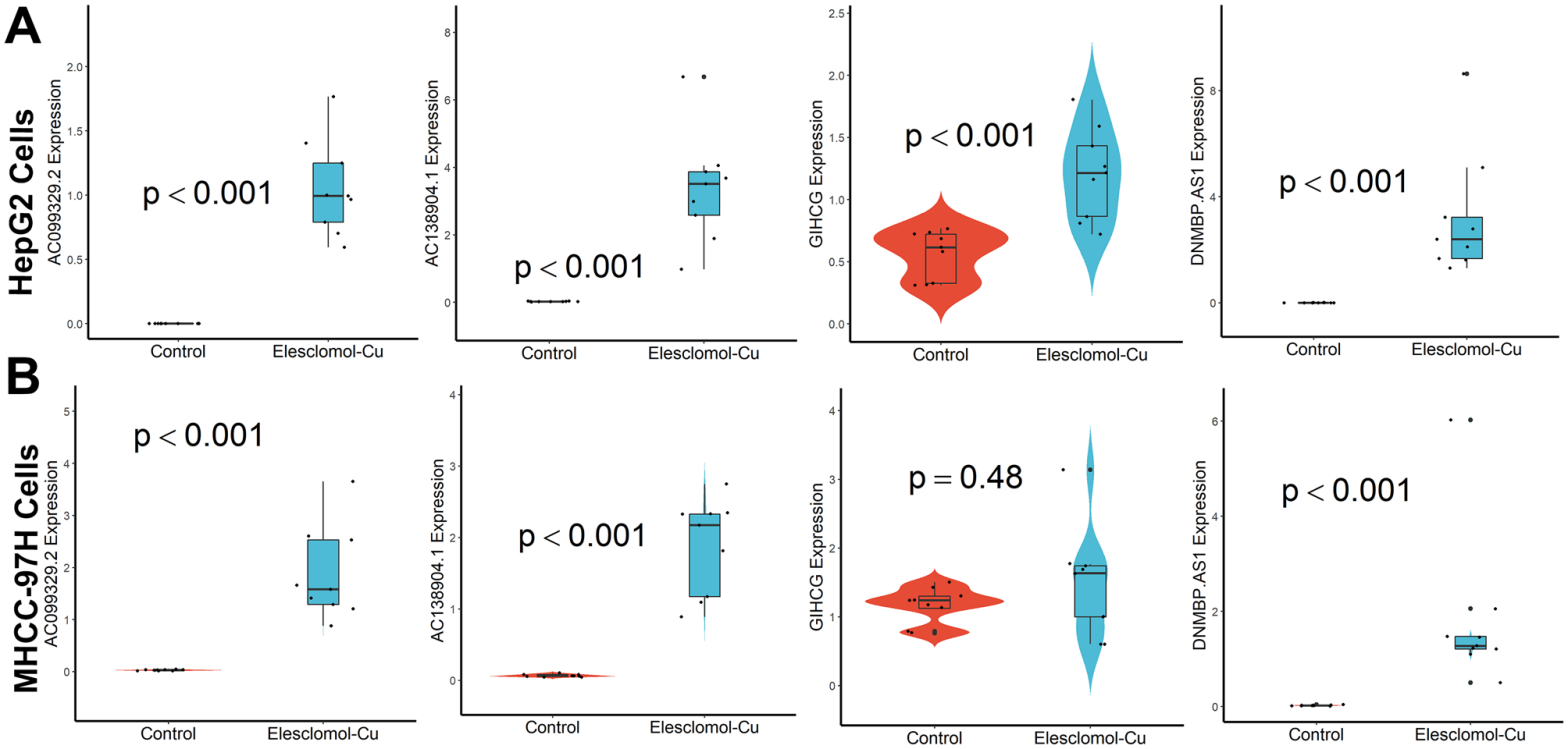
qRT-PCR assay of the lncRNAs expression levels in the constructed Cuproptosis cell model. (**A**) In HerpG2 cells, (**B**) In MHCC-97H cells.

**Figure 11:**
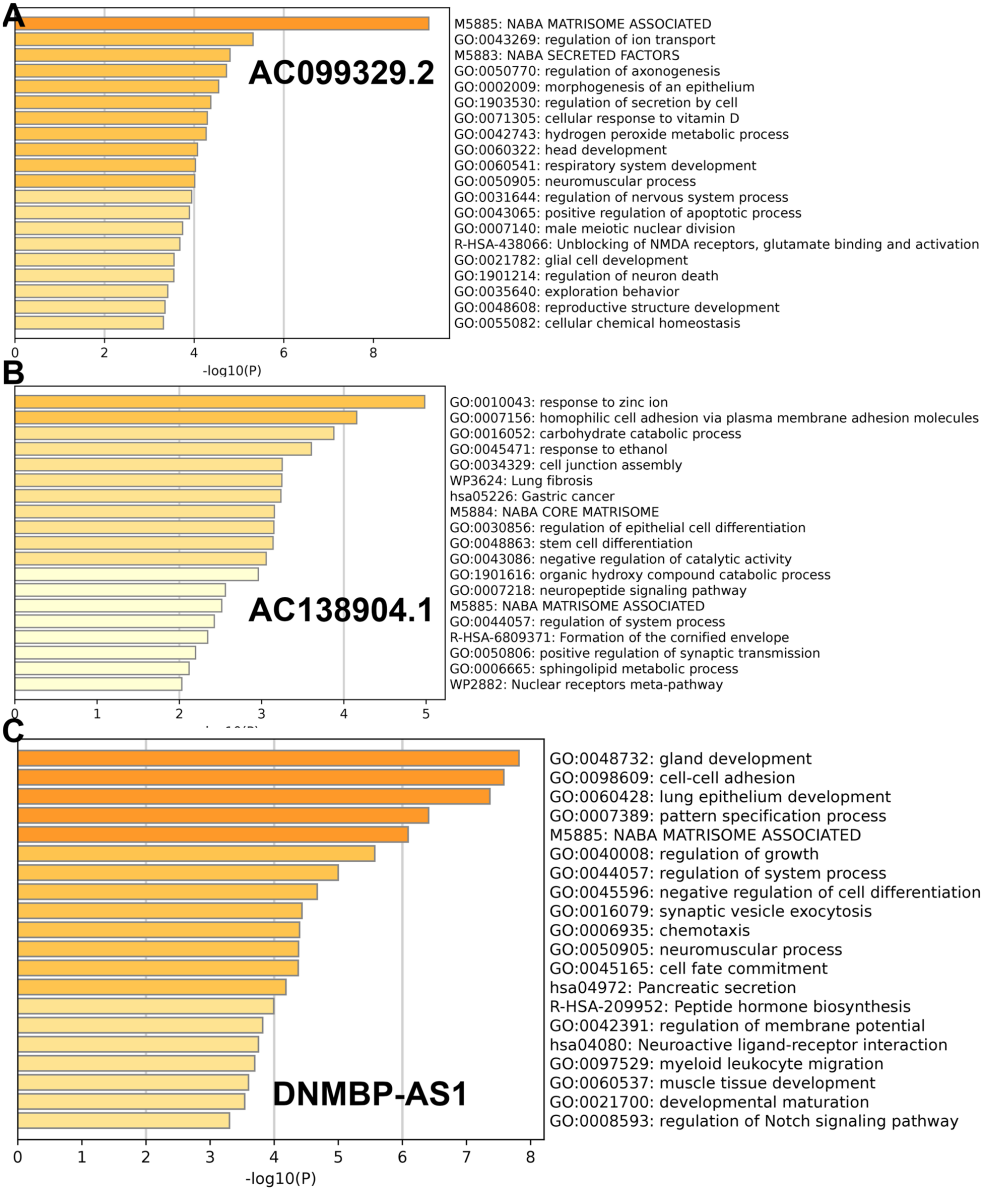
Functional enrichment analysis of lncRNAs. (**A**) AC099329.2. (**B**) AC138904.1. (**C**) DNMBP-AS1.

As shown in Fig. 10A, the expression of AC138904.1, AC099329.2, GIHCG, and DNMBP-AS1 were significantly changed before and after drug treatment in HerpG2 cells. However, in MHCC-97H cells, only the expression of AC138904.1, AC099329.2, and DNMBP-AS1 were significantly changed (Fig. 10B). Unfortunately, DEPDC1-AS1 and AC145343.1 were not detected in either cell line, probably due to being cell-specific or under-expressed. Finally, we explored the potential mechanisms of the three lncRNAs identified above in HCC by GSEA and found that all of them were related to the regulation of ion transport (Fig. 11).

## Discussion

Although appropriate concentrations of copper are involved in a variety of metabolic processes, including energy production, peptide amination, synthesis of catecholamines, iron ion transport, superoxide dismutation, and extracellular matrix biosynthesis, it can be toxic at elevated concentrations. A growing number of studies have shown that copper homeostasis is closely associated with the development of a variety of tumors and that cytotoxicity caused by its imbalance can regulate the rate of cancer cell growth and proliferation^19^. An in-depth exploration of the mechanism of increased intracellular toxicity due to an imbalance in copper homeostasis caused by copper ion accumulation could help us to efficiently and selectively kill cancer cells in immunotherapy^20^. Excitingly, Tsvetkov found in his research that copper can cause aggregation of lipid acylated proteins and loss of iron-sulfur (Fe-S) cluster proteins and increase proteotoxic stress by directly binding the lipid acylated components of the tricarboxylic acid (TCA) cycle, ultimately leading to cell death^10^. This novel form of cell death is defined as Cuproptosis, which is completely different from known forms of cell death such as pyroptosis, apoptosis, ferroptosis, and necroptosis. Considering that lipid acylation and Fe-S cluster proteins are widely and conservatively present in nature as the main targets of cytotoxicity produced by Cuproptosis, therapeutic options for copper ions targeting tumors with this metabolic profile are promising, and biomarkers that can reliably and precisely detect Cuproptosis in complicated human tumor tissues is urgently needed.

Ten genes (including FDX1, LIPT1, DLD, LIAS, DLAT, PDHA1, PDHB, MTF1, GLS, and CDKN2A) were obtained from a previous study^10^ and were considered Cuproptosis-related genes. FDX1 gene codes for a tiny iron-sulfur protein that transports electrons from NADPH to mitochondrial cytochrome P450 via ferredoxin reductase, which is implicated in the steroid, vitamin D, and bile acid metabolism. FDX1 may affect the prognosis of lung adenocarcinoma by mediating metabolism^21^. Lipoyltransferase 1 (LIPT1) is an enzyme that activates TCA cycle-associated 2-ketoacid dehydrogenases, and its deficiency inhibits the metabolism of the TCA cycle^22^. Dihydrolipamine dehydrogenase (DLD) was reported to modulate cysteine deprivation-induced ferroptosis^23^. Deletion of FDX1 and LIAS inhibited the occurrence of Cuproptosis^10^. Over-expressed DLAT could promote gastric cancer cell proliferation by regulating carbohydrate metabolism^24^. Aberrant expression of PDHA1 and PDHB, which were involved in glycolytic regulation, was also closely associated with poor prognosis in gastric cancer patients^25–27^. Metal regulatory transcription factor 1 (MTF1) is a metal-binding transcription factor found in eukaryotes that protects cells from oxidative and hypoxic stress by responding to both metal excess and deficiency, and its deletion could inhibit the epithelial to mesenchymal transition^28^. Glutaminase (GLS) is a key enzyme in glutamine metabolism and has been reported to be involved in several types of cancer^29,30^. CDKN2A was reported to be a tumor suppressor gene on chromosome 9p21.3 that played a role in tumor suppression of tumor proliferation^31^. However, CDKN2A expression was upregulated in HCC and was strongly associated with poor patient prognosis^32^. In addition, CDKN2A methylation levels were also associated with copper ion metabolism in humans^33^. These all suggested the involvement of Cuproptosis-related genes in the prognostic development of cancer.

Following the discovery of DE-lncRNA and Cuproptosis-associated lncRNAs, a personalized prognostic profile was created in this work using overlapping lncRNAs that can be used to predict treatment response to ICB therapy and are also considered an independent predictor of HCC prognosis. Furthermore, comparing the prediction potential of several genetic markers can help researchers learn more about their prognostic significance. As a result, we compared the prediction capacity of the six gene signatures shown below: (1) the Cuproptosis-associated lncRNA signature constructed in this study; (2) the ferroptosis-related nine-lncRNA signature constructed by Xu et al.^13^; (3) the twelve immune-related lncRNA signature constructed by Hong et al.^34^; (4) the eleven lncRNA signature constructed by Wang et al.^35^ and (5) the eight m6A methyltransferase-related lncRNAs constructed by Li et al.^12^; (6) the eight ferroptosis- and immune-related lncRNA signature constructed by Huang et al.^36^; (7) the fourteen pyroptosis-Related lncRNAs signature and the five ferroptosis-related lncRNA signature constructed by Tang et al.^37,38^. The results showed that although all the above signatures were more accurate predictors of patient prognosis, our six Cuproptosis-associated lncRNA signature contained the least number of genes and was relatively easy to apply clinically when compared to other prior signatures. Moreover, based on the GSEA enrichment results we found that the Cuproptosis-associated lncRNA risk model we constructed in our study was not only related to the metabolism and transport of copper ions, but also cell proliferation, cell adhesion, cell metabolism, and cell cycle regulation. Finally, Cuproptosis was also closely related to the tumor immune microenvironment. Both the TIMER and XCELL results showed significantly higher levels of CD4^+^ T cell infiltration in the high-risk scoring group, suggesting that Cuproptosis may be involved in the regulation of HCC progression through increased levels of CD4^+^ T cell infiltration. Of course, this needs to be verified by detailed experiments in vivo and in vitro at a later stage.

Among the six lncRNA involved in our signature, AC099329.2 and DNMBP-AS1 were protective lncRNA in HCC patients, while AC138904.1, DEPDC1-AS1, GIHCG, and AC145343.1 were risk lncNRAs. AC099329.2 and DNMBP-AS1 have been reported to be strongly associated with ferroptosis and could be used as a novel biomarker for predicting Breast Cancer prognosis^39,40^, and DEPDC1-AS1 has also been shown to be significantly correlated with ferroptosis and may serve as a good prognostic marker for lung adenocarcinoma^41,42^. However, their functions and mechanisms of them have not been reported so far in HCC. AC145343.1 can be involved in the regulation of immune cell infiltration as TP53 mutation-associated lncRNA and predict HCC patient prognosis^43,44^. High GIHCG expression was linked to a shorter patient survival time and was also an independent predictive factor for overall survival in HCC^45^, which was in line with our findings. Knocking down GIHCG dramatically suppressed HCC cell growth, proliferation, and metastasis in vitro and in vivo, and was predicted to be a novel target for HCC treatment^46^. Moreover, a high level of GIHCG expression was also a biomarker for Lapatinib resistance^47^. GIHCG has also been shown to be involved in the development of a variety of tumors including breast^48^, ovarian^49^, cervical^50^, esophageal^51^, colorectal^52^, and gastric^53^ cancers. In this study, we found that the expression of AC138904.1, AC099329.2, and DNMBP-AS1 was significantly changed before and after drug treatment in both HerpG2 and MHCC-97H cells in the constructed Cuproptosis cell model and might be involved in the development of HCC through the mechanism of Cuproptosis, and further studies are needed to confirm this result.

There is no doubt that our study has certain limitations. First, individual variations in HCC patients may influence our Cuproptosis-related lncRNA signature, which has only been evaluated in the TCGA dataset. Future external and practical testing will be required to establish if it can be applied to clinical patients. Furthermore, we have only a limited understanding of the signaling pathways involved in Cuproptosis-related lncRNAs; however, the specific molecular mechanisms of the six lncRNAs in HCC, as well as their interactions with TME and Cuproptosis, remain unknown, and their roles must be investigated in vivo and in vitro using GSEA results as a guide in the future.

## Conclusions

Therefore, we created a prognostic six-lncRNA profile linked with Cuproptosis to predict immunotherapy treatment response, which may bring new insights into the molecular pathways underlying HCC formation and progression (Supplementary Information).

## Supplementary Information

Supplementary Information.

## Data Availability

The datasets used and/or analyzed during the current study are available from the corresponding author on reasonable request.

## References

[1] Kim BE, Nevitt T, Thiele DJ (2008). Mechanisms for copper acquisition, distribution and regulation. Nat. Chem. Biol..

[2] Lutsenko S (2010). Human copper homeostasis: A network of interconnected pathways. Curr. Opin. Chem. Biol..

[3] Bandmann O, Weiss KH, Kaler SG (2015). Wilson's disease and other neurological copper disorders. Lancet Neurol..

[4] Gaggelli E, Kozlowski H, Valensin D, Valensin G (2006). Copper homeostasis and neurodegenerative disorders (Alzheimer's, prion, and Parkinson's diseases and amyotrophic lateral sclerosis). Chem. Rev..

[5] Atakul T, Altinkaya SO, Abas BI, Yenisey C (2020). Serum copper and zinc levels in patients with endometrial cancer. Biol. Trace Elem. Res..

[6] Ressnerova A, Raudenska M, Holubova M, Svobodova M, Polanska H, Babula P, Masarik M, Gumulec J (2016). Zinc and copper homeostasis in head and neck cancer: Review and meta-analysis. Curr. Med. Chem..

[7] Ge EJ, Bush AI, Casini A, Cobine PA, Cross JR, DeNicola GM, Dou QP, Franz KJ, Gohil VM, Gupta S (2022). Connecting copper and cancer: From transition metal signalling to metalloplasia. Nat. Rev. Cancer.

[8] da Silva DA, De Luca A, Squitti R, Rongioletti M, Rossi L, Machado CML, Cerchiaro G (2022). Copper in tumors and the use of copper-based compounds in cancer treatment. J. Inorg. Biochem..

[9] Li H, Wang J, Wu C, Wang L, Chen ZS, Cui W (2020). The combination of disulfiram and copper for cancer treatment. Drug Discov. Today.

[10] Tsvetkov P, Coy S, Petrova B, Dreishpoon M, Verma A, Abdusamad M, Rossen J, Joesch-Cohen L, Humeidi R, Spangler RD (2022). Copper induces cell death by targeting lipoylated TCA cycle proteins. Science.

[11] Zhou C, Zhang H, Lu L (2021). Identification and validation of hypoxia-related lncRNA signature as a prognostic model for hepatocellular carcinoma. Front. Genet..

[12] Li, L., Xie, R. & Lu, G. Identification of m6A methyltransferase-related lncRNA signature for predicting immunotherapy and prognosis in patients with hepatocellular carcinoma. *Biosci. Rep.* **41**(6), BSR20210760 (2021). 10.1042/BSR20210760 PMC8188173 34027555

[13] Xu Z, Peng B, Liang Q, Chen X, Cai Y, Zeng S, Gao K, Wang X, Yi Q, Gong Z (2021). Construction of a ferroptosis-related nine-lncRNA signature for predicting prognosis and immune response in hepatocellular carcinoma. Front. Immunol..

[14] Yang S, Zhou Y, Zhang X, Wang L, Fu J, Zhao X, Yang L (2021). The prognostic value of an autophagy-related lncRNA signature in hepatocellular carcinoma. BMC Bioinform..

[15] Bai Y, Lin H, Chen J, Wu Y, Yu S (2021). Identification of prognostic glycolysis-related lncRNA signature in tumor immune microenvironment of hepatocellular carcinoma. Front. Mol. Biosci..

[16] Zhou Y, Zhou B, Pache L, Chang M, Khodabakhshi AH, Tanaseichuk O, Benner C, Chanda SK (2019). Metascape provides a biologist-oriented resource for the analysis of systems-level datasets. Nat. Commun..

[17] Li T, Fan J, Wang B, Traugh N, Chen Q, Liu JS, Li B, Liu XS (2017). TIMER: A web server for comprehensive analysis of tumor-infiltrating immune cells. Can. Res..

[18] Aran D, Hu Z, Butte AJ (2017). xCell: Digitally portraying the tissue cellular heterogeneity landscape. Genome Biol..

[19] Shanbhag VC, Gudekar N, Jasmer K, Papageorgiou C, Singh K, Petris MJ (2021). Copper metabolism as a unique vulnerability in cancer. Biochim. Biophys. Acta.

[20] Kahlson MA, Dixon SJ (2022). Copper-induced cell death. Science.

[21] Zhang Z, Ma Y, Guo X, Du Y, Zhu Q, Wang X, Duan C (2021). FDX1 can impact the prognosis and mediate the metabolism of lung adenocarcinoma. Front. Pharmacol..

[22] Solmonson A, Faubert B, Gu W, Rao A, Cowdin MA, Menendez-Montes I, Kelekar S, Rogers TJ, Pan C, Guevara G (2022). Compartmentalized metabolism supports midgestation mammalian development. Nature.

[23] Shin D, Lee J, You JH, Kim D, Roh JL (2020). Dihydrolipoamide dehydrogenase regulates cystine deprivation-induced ferroptosis in head and neck cancer. Redox Biol..

[24] Goh WQ, Ow GS, Kuznetsov VA, Chong S, Lim YP (2015). DLAT subunit of the pyruvate dehydrogenase complex is upregulated in gastric cancer-implications in cancer therapy. Am. J. Transl. Res..

[25] Song L, Liu D, Zhang X, Zhu X, Lu X, Huang J, Yang L, Wu Y (2019). Low expression of PDHA1 predicts poor prognosis in gastric cancer. Pathol. Res. Pract..

[26] Liu Z, Yu M, Fei B, Fang X, Ma T, Wang D (2018). miR-21-5p targets PDHA1 to regulate glycolysis and cancer progression in gastric cancer. Oncol. Rep..

[27] Cai Z, Zhao JS, Li JJ, Peng DN, Wang XY, Chen TL, Qiu YP, Chen PP, Li WJ, Xu LY (2010). A combined proteomics and metabolomics profiling of gastric cardia cancer reveals characteristic dysregulations in glucose metabolism. Mol. Cell. Proteomics.

[28] Ji L, Zhao G, Zhang P, Huo W, Dong P, Watari H, Jia L, Pfeffer LM, Yue J, Zheng J (2018). Knockout of MTF1 inhibits the epithelial to mesenchymal transition in ovarian cancer cells. J. Cancer.

[29] Masisi BK, El Ansari R, Alfarsi L, Rakha EA, Green AR, Craze ML (2020). The role of glutaminase in cancer. Histopathology.

[30] Matés JM, Campos-Sandoval JA, Santos-Jiménez JL, Márquez J (2019). Dysregulation of glutaminase and glutamine synthetase in cancer. Cancer Lett..

[31] Zhao R, Choi BY, Lee MH, Bode AM, Dong Z (2016). Implications of genetic and epigenetic alterations of CDKN2A (p16(INK4a)) in cancer. EBioMedicine.

[32] Luo, J. P., Wang, J. & Huang, J. H. CDKN2A is a prognostic biomarker and correlated with immune infiltrates in hepatocellular carcinoma. *Biosci. Rep.* **41**(10), BSR20211103 (2021). 10.1042/BSR20211103 PMC8495430 34405225

[33] Silva IR, Francisco LFV, Bernardo C, Oliveira MA, Barbosa F, Silveira HCS (2020). DNA methylation changes in promoter region of CDKN2A gene in workers exposed in construction environment. Biomarkers.

[34] Hong W, Liang L, Gu Y, Qi Z, Qiu H, Yang X, Zeng W, Ma L, Xie J (2020). Immune-related lncRNA to construct novel signature and predict the immune landscape of human hepatocellular carcinoma. Mol. Ther. Nucl. Acids.

[35] Li W, Chen QF, Huang T, Wu P, Shen L, Huang ZL (2020). Identification and validation of a prognostic lncRNA signature for hepatocellular carcinoma. Front. Oncol..

[36] Huang A, Li T, Xie X, Xia J (2021). Computational identification of immune- and ferroptosis-related LncRNA signature for prognosis of hepatocellular carcinoma. Front. Mol. Biosci..

[37] Tang X, Zhang A, Feng Y, Su Y, Wang X, Jiang F, Ma J (2021). A novel pyroptosis-related lncRNAs signature for predicting the prognosis of kidney renal clear cell carcinoma and its associations with immunity. J. Oncol..

[38] Tang X, Jiang F, Wang X, Xia Y, Mao Y, Chen Y (2022). Identification of the ferroptosis-related long non-coding RNAs signature to improve the prognosis prediction in papillary renal cell carcinoma. Front. Surg..

[39] Xu Z, Jiang S, Ma J, Tang D, Yan C, Fang K (2021). Comprehensive analysis of ferroptosis-related LncRNAs in breast cancer patients reveals prognostic value and relationship with tumor immune microenvironment. Front. Surg..

[40] Gao S, Lu X, Ma J, Zhou Q, Tang R, Fu Z, Wang F, Lv M, Lu C (2021). Comprehensive analysis of lncRNA and miRNA regulatory network reveals potential prognostic non-coding RNA involved in breast cancer progression. Front. Genet..

[41] Lu L, Liu LP, Zhao QQ, Gui R, Zhao QY (2021). Identification of a ferroptosis-related LncRNA signature as a novel prognosis model for lung adenocarcinoma. Front. Oncol..

[42] Yang L, Wu Y, Xu H, Zhang J, Zheng X, Zhang L, Wang Y, Chen W, Wang K (2021). Identification and validation of a novel six-lncRNA-based prognostic model for lung adenocarcinoma. Front. Oncol..

[43] Wu J, Ren X, Wang N, Zhou R, Chen M, Cai Y, Lin S, Zhang H, Xie X, Dang C (2021). A mutation-related long noncoding RNA Signature of genome instability predicts immune infiltration and hepatocellular carcinoma prognosis. Front. Genet..

[44] Huang DP, Liao MM, Tong JJ, Yuan WQ, Peng DT, Lai JP, Zeng YH, Qiu YJ, Tong GD (2021). Construction of a genome instability-derived lncRNA-based risk scoring system for the prognosis of hepatocellular carcinoma. Aging.

[45] Xiao S, Huang S, Yang J (2020). Overexpression of GIHCG is associated with a poor prognosis and immune infiltration in hepatocellular carcinoma. Onco. Targets. Ther..

[46] Sui CJ, Zhou YM, Shen WF, Dai BH, Lu JJ, Zhang MF, Yang JM (2016). Long noncoding RNA GIHCG promotes hepatocellular carcinoma progression through epigenetically regulating miR-200b/a/429. J. Mol. Med..

[47] Xiang Z, Song S, Zhu Z, Sun W, Gifts JE, Sun S, Li QS, Yu Y, Li KK (2019). LncRNAs GIHCG and SPINT1-AS1 are crucial factors for pan-cancer cells sensitivity to lapatinib. Front. Genet..

[48] Fan LY, Shi KY, Xu D, Ren LP, Yang P, Zhang L, Wang F, Shao GL (2019). LncRNA GIHCG regulates microRNA-1281 and promotes malignant progression of breast cancer. Eur. Rev. Med. Pharmacol. Sci..

[49] Yao N, Yu L, Zhu B, Gan HY, Guo BQ (2018). LncRNA GIHCG promotes development of ovarian cancer by regulating microRNA-429. Eur. Rev. Med. Pharmacol. Sci..

[50] Zhang X, Mao L, Li L, He Z, Wang N, Song Y (2019). Long noncoding RNA GIHCG functions as an oncogene and serves as a serum diagnostic biomarker for cervical cancer. J. Cancer.

[51] Zhao W, Huang Z, Liu H, Wang C (2020). LncRNA GIHCG promotes the development of esophageal cancer by modulating miR-29b-3p/ANO1 Axis. Onco. Targets. Ther..

[52] Jiang X, Li Q, Zhang S, Song C, Zheng P (2019). Long noncoding RNA GIHCG induces cancer progression and chemoresistance and indicates poor prognosis in colorectal cancer. Onco. Targets. Ther..

[53] Liu G, Jiang Z, Qiao M, Wang F (2019). Lnc-GIHCG promotes cell proliferation and migration in gastric cancer through miR-1281 adsorption. Mol. Genet. Genomic Med..

